# “The information exists, but it's your job to find it”: 1.5-generation immigrants' encounters with public institutions

**DOI:** 10.3389/fsoc.2026.1857620

**Published:** 2026-10-01

**Authors:** Pnina Dolberg, Karin Amit, Avital Kaye-Tzadok

**Affiliations:** 1 Department of Social Work, Ruppin Academic Center, Emek Hefer, Israel; 2 The Institute of Immigration and Social Integration, Ruppin Academic Center, Emek Hefer, Israel; 3 Faculty of Management and Economics, Ruppin Academic Center, Emek Hefer, Israel; 4 The Interdisciplinary Research Center for Advancing Mental and Social Health, Ruppin Academic Center, Emek Hefer, Israel

**Keywords:** immigrant satisfaction, public institutions, institutional encounters, retrospective interpretation, 1.5 generation immigrants

## Abstract

Public institutions play a central role in immigrants' access to rights, resources, and opportunities, yet less is known about how immigrants experience encounters across multiple institutional settings during the early years following migration. Based on qualitative interviews with 21 immigrants from the former Soviet Union to Israel, this study examines how adults who immigrated during adolescence (referred to as the 1.5 generation) retrospectively describe and interpret their early interactions with public institutions. We identify four central themes: absence of institutional guidance, institutional misfit and misrecognition, active mediation by immigrant youth within their families, and localized experiences of support within specific institutional contexts. Participants' accounts point to a recurring gap between the formal availability of institutional resources and participants' ability to identify, access, and use those resources in practice. Information, guidance, mediation, recognition, and cultural sensitivity shaped how they experienced institutional encounters, while adolescents often became central intermediaries between their families and public systems. Positive experiences were commonly associated with particular institutional actors or structured programs that provided support and opportunities. The study highlights the value of examining early institutional encounters from a broader vantage point of adulthood.

## Introduction

Immigrants' everyday interactions with state institutions constitute a central arena where access to resources is mediated, social positions negotiated, and relationships with the host society formed (Gal, 2008; Kogan and Shen, 2019). Encounters with systems such as education, welfare, and healthcare play a formative role in shaping immigrants' experiences of and positioning within the host society. This is particularly the case for those who immigrate during adolescence, when social identities are formed and patterns of engagement with institutional environments begin to take shape (Suárez-Orozco et al., 2009).

Research has examined how immigrants' satisfaction with public services and institutions is shaped by everyday interactions with institutional actors and by perceptions of accessibility, fairness, and responsiveness (Kogan and Shen, 2019; Röder and Mühlau, 2012). Most studies have focused on how individuals experience and evaluate institutional encounters in relation to service use at the time they occur. Less attention has been paid to how immigrants make sense of their early encounters with public institutions across different institutional settings, particularly when viewed from a broader temporal perspective (Eastmond, 2007). To address this gap, the present study draws on retrospective interviews with adults who immigrated from the former Soviet Union (FSU) to Israel during adolescence.

### Satisfaction with institutions and public services

Citizen satisfaction has been defined by Kim et al. (2024, p. 468) as an “evaluation based on one's experience with a public organization or public service.” At its core is a judgment from the citizen's perspective of the quality of services received. Citizen satisfaction has been explained through antecedent factors: environmental (community and national-level), organizational, and individual (Kim et al., 2024). Some debate persists regarding its definition, measurement, and underlying theoretical mechanisms (Li et al., 2026). Citizen satisfaction is an outcome in itself and is closely linked to broader attitudinal constructs, particularly trust in government at both local and national levels (Kim et al., 2024).

Modern societies are sustained by abstract systems, encompassing large-scale institutional arrangements, such as the nation-state, and more specialized professional domains, including welfare and healthcare. Trust in such systems is commonly understood as confidence in their reliability with regard to specific outcomes, reflecting belief in both the system's integrity and the validity of its underlying principles or technical knowledge (Giddens, 1991). Trust in abstract systems is shaped primarily at their “access points,” the organizational sites of direct encounter, where service users and welfare institutions interact. Here the otherwise “faceless” system becomes concrete and experientially accessible (Giddens, 1991). Through these encounters, trust can be strengthened, maintained, or eroded, depending on how individuals interpret their interactions with institutional representatives (Yang and Holzer, 2006). The relationship between institutional performance and trust can be understood through individuals' experiences with public services. Evaluations of service quality and satisfaction contribute to the formation of institutional trust (Van de Walle and Bouckaert, 2003). Trust emerges as a relational and processual outcome, shaped through ongoing interactions and experience-based evaluations of institutional performance (Christensen and Lægreid, 2005; Van de Walle and Bouckaert, 2003).

### Immigrants' experiences and evaluations of public institutions

Research on immigrants' satisfaction has largely focused on overall life satisfaction and subjective wellbeing as indicators of successful integration into host societies. Within this literature, life satisfaction is typically conceptualized as a broad quality evaluation, shaped by socioeconomic integration, labor market participation, social inclusion, and experiences of discrimination (De Vroome and Hooghe, 2014; Paloma et al., 2021). While this body of research provides important insights into immigrants' wellbeing, it primarily addresses general evaluations, with less attention to assessments of specific institutional encounters. Yet interactions with public institutions, through which immigrants access rights, resources, and opportunities, are likely to shape broader perceptions of integration and wellbeing. These encounters therefore constitute an important dimension of immigrants' broader evaluations of the receiving society.

Satisfaction with public institutions constitutes a central dimension of immigrants' experiences. It may be shaped by their perceptions of accessibility, fairness, responsiveness, and legitimacy. Satisfaction reflects evaluations of both service effectiveness and the broader quality of state–immigrant relations. Evaluations are often grounded in immigrants' assessments of how key domains of the host society (the economy, democratic institutions, and the provision of public goods such as education and healthcare) function in practice (Kogan and Shen, 2019). Their perceptions reflect both objective institutional conditions and subjective evaluations shaped by expectations, needs, and prior experiences. Repeated experiences of satisfaction or dissatisfaction may accumulate over time and influence broader levels of institutional trust and perceived legitimacy (Quaranta, 2025; Röder and Mühlau, 2012).

Empirical research shows that immigrant satisfaction is strongly shaped by everyday encounters with public institutions (Kogan and Shen, 2019). Interactions with frontline actors, such as social workers, teachers, healthcare providers, and local administrators, often constitute immigrants' most direct contact with the state. These encounters provide opportunities to evaluate whether institutions are accessible, transparent, and responsive to their needs. Fersch and Breidahl (2018) demonstrate that migrants' trust in the Danish welfare state is shaped through interactions with welfare institutions: experiences of fairness and responsiveness strengthen institutional confidence, whereas bureaucratic obstacles undermine it. Similarly, research on forced migrants in the Finnish welfare state shows that trust and satisfaction are continuously negotiated through institutional encounters, as migrants assess the transparency and predictability of institutional procedures (Sundbäck, 2025). These findings suggest that evaluations are shaped through experiences of institutional processes as well as their outcomes.

Another important factor shaping immigrant satisfaction concerns perceptions of recognition and equal treatment. Belabas and Migchelbrink (2025) show that trust in local government is influenced by experiences of discrimination, perceptions of responsiveness, and recognition from public authorities. Similarly, Tyrberg (2024) demonstrates that experiences of fair treatment and support can strengthen immigrants' sense of belonging, whereas perceived discrimination undermines both trust and satisfaction. These findings highlight the symbolic dimension of institutional encounters, as signaling to immigrants whether they are recognized as legitimate social and political community members.

In the present study, institutional evaluation is used as a broad term for participants' assessments of their encounters with public institutions. Consistent with previous research, satisfaction refers to evaluations of particular services or institutional experiences, while trust refers to broader confidence in the reliability and integrity of institutional systems (Giddens, 1991; Kim et al., 2024; Van de Walle and Bouckaert, 2003). Responsiveness and recognition are treated as dimensions of institutional encounters that may shape these evaluations, including whether institutions are perceived as addressing immigrants' needs and acknowledging their identities, capacities, and experiences (Belabas and Migchelbrink, 2025; Fersch and Breidahl, 2018).

### Immigrants' experiences of public institutions in Israel

Israel is a society shaped by immigration (Amit et al., 2010; Central Bureau of Statistics, 2020). Historically, Jewish immigration (Aliyah) has been framed in terms of return. Since the establishment of the state, Israel has adopted an open-door policy granting immediate citizenship and extensive social rights to Jewish immigrants and their descendants (Lewin-Epstein and Semyonov, 2013). The state is actively involved in immigrant absorption, providing financial support, housing assistance, and access to language and employment training programs (Semyonov et al., 2015). As argued by Gal (2008), Israel's welfare state allocates social rights through institutional categories closely linked to immigration status and national belonging. Policies are implemented through a wide range of public institutions, including welfare, education, and healthcare systems, which structure access to resources and opportunities (Gal, 2008; Kagan, 2021).

Against this background, immigrants' satisfaction with public institutions in Israel must be understood within a highly stratified welfare state, where access to rights and services is shaped by immigration status, ethnicity, and institutional classifications (Gal, 2008). Research on immigrants in Israel highlights both structural constraints and experiential dimensions of institutional engagement. Pinchas-Mizrachi et al. (2020) demonstrate that trust in the Israeli healthcare system varies across groups, with immigrants often reporting lower trust levels, reflecting disparities in perceived fairness and responsiveness. More recent work suggests that institutional trust among immigrants is linked to their capacity for economic integration and entrepreneurial activity (Gurevich and Kushnirovich, 2025). A study of FSU immigrants in Israel by Kagan (2021) shows that patterns of social service utilization are shaped by formal eligibility, perceived accessibility, cultural fit, and prior experiences with state institutions.

Research on FSU immigrants in Israel also highlights the importance of pre-migration institutional orientations in shaping expectations of public institutions after migration. Knaifel (2022) describes Soviet socialization as fostering ambivalent attitudes toward state institutions and public authorities, combining suspicion with dependence on external authority. Remennick (2015) similarly emphasizes the persistence of shared outlooks and institutional orientations shaped by Soviet and post-Soviet experience, while also describing a receiving context in which extensive rights granted upon arrival were accompanied by expectations of rapid adaptation to Israeli social and cultural norms.

### FSU immigration and integration in Israel

Beginning in the late 1980s and continuing after the collapse of the Soviet Union, more than one million immigrants arrived in Israel during the 1990s and early 2000s, constituting Israel's largest immigrant group (Central Bureau of Statistics, 2020; Remennick, 2007). While often treated as a single entity, FSU immigrants are a heterogeneous population, differing in country of origin, ethnic and cultural background, attachment to Jewish identity, historical experience, and patterns of integration (Knaifel, 2022; Konstantinov, 2015). The term FSU is therefore used here not to imply cultural or institutional homogeneity, but to refer to a historically situated migration population whose members were part of the large migration wave to Israel during the late Soviet and post-Soviet transition. Despite their diverse republics and social backgrounds, members of this historical cohort shared, to varying degrees, experiences, memories, and orientations shaped by Soviet or post-Soviet socialization (Knaifel, 2022; Remennick, 2015). They were also characterized, at the aggregate level, by relatively high levels of education and professional training prior to arrival (Central Bureau of Statistics, 2016; Remennick, 2007).

Despite these advantages, the integration of FSU immigrants has been marked by diverse experiences and persistent challenges. Many immigrants experienced difficulties transferring their professional credentials to the Israeli labor market, leading to widespread occupational downgrading, particularly in the early years (Dolberg et al., 2019; Remennick, 2003). Moreover, early patterns of residential concentration and reliance on Russian-speaking social networks suggest that initial stages of integration were marked by partial social separation (Raijman and Kemp, 2010). Experiences of stereotyping and discrimination further shaped immigrants' incorporation into Israeli society (Remennick and Prashizky, 2019).

Over time, patterns of integration have evolved. Research indicates gradual improvements in Hebrew language proficiency, employment outcomes, and overall socioeconomic status, alongside a growing sense of belonging among many immigrants (Amit, 2012; Central Bureau of Statistics, 2016; Dolberg et al., 2016; Konstantinov, 2015). At the same time, cultural distinctiveness and diverse integration experiences have persisted across subgroups.

### 1.5 Generation, adolescence, and early institutional encounters

Immigrants who arrive in the host country during childhood or adolescence are commonly referred to as the “1.5 generation,” reflecting their intermediate position between first- and second-generation immigrants (Dolberg and Amit, 2023; Rumbaut, 2004; Rumbaut and Ima, 1988). Having been socialized across both origin and host societies, they develop familiarity with dual cultural frameworks, often experiencing a sense of partial belonging to each (Remennick and Prashizky, 2019; Rumbaut and Ima, 1988). This positioning shapes identity formation and patterns of engagement with social institutions (Katsiaficas, 2017).

A central feature of the 1.5 generation experience is their early and intensive engagement with core societal systems, particularly education. Schools are where immigrant youth encounter the host society, acquire language, form social networks, and are exposed to dominant norms and expectations (Suárez-Orozco et al., 2009). However, research shows that schools may also be sites of exclusion, stereotyping, and boundary-making, particularly for immigrant youth navigating linguistic and cultural differences (Dolberg and Amit, 2023; Remennick, 2003). In the Israeli context, qualitative evidence indicates that many 1.5 generation immigrants experienced social segregation and discrimination during their school years, shaping their early positioning within the host society (Dolberg and Amit, 2023; Remennick and Prashizky, 2019).

Beyond their own institutional experiences, 1.5 generation immigrants often assume a mediating role between their families and host-society institutions. Due to their relatively rapid acquisition of language and cultural competencies, they frequently serve as cultural and linguistic brokers for their parents in interactions with public systems. This role places them in a position of early responsibility and sustained engagement with institutional structures (Katsiaficas, 2017; Suárez-Orozco et al., 2009).

Research suggests that early institutional experiences during childhood and adolescence may remain significant beyond the initial period of immigration. In the Israeli case, studies show that initial experiences of exclusion and marginalization may coexist with processes of integration and identity transformation over time (Dolberg and Amit, 2023; Remennick and Prashizky, 2019).

### Retrospective perspective as an analytical lens

A retrospective perspective offers a useful lens for understanding how immigrants interpret their early encounters with state institutions. Such accounts provide access to early experiences as they are remembered and understood from the perspective of adulthood, informed by subsequent experiences and later knowledge.

In immigration contexts, where individuals often undergo multiple personal, social, and institutional transitions, this lens makes it possible to examine how early institutional experiences are later interpreted (Eastmond, 2007).

### The present study

Despite extensive research on immigrant integration and encounters with public institutions, the literature has primarily examined institutional engagement in relation to access, service use, and short-term outcomes. Although research has examined the experiences of 1.5-generation immigrants in specific institutional settings, particularly schools, much less is known about their early experiences across multiple public institutions during the initial years following immigration. This gap is important because public institutions are among immigrants' main points of contact with the receiving state and shape access to rights, resources, and opportunities during the early years after immigration. Against this backdrop, the present study examines how adults who immigrated during adolescence (the 1.5 generation) retrospectively describe and interpret their early encounters with public institutions.

## Method

This study employed a qualitative-inductive approach to explore how immigrants interpret their early encounters with public institutions, drawing on participants' subjective meanings and retrospective accounts of early institutional experiences. Research was conducted within a constructivist framework, emphasizing interpretation of lived experiences in their social and temporal context (Padgett, 2017). Reporting follows the APA JARS-Qual guidelines (Levitt et al., 2018).

### Participants

The sample comprised 21 participants (12 women, nine men) who immigrated to Israel from the FSU during early to late adolescence. Inclusion criteria were immigration to Israel from the FSU between ages 12 and 18, and willingness to participate in an in-depth interview. Participants were born between 1976 and 1988 and arrived between the ages of 12 and 17.5.

Participants varied in sociodemographic characteristics, including country of origin (Ukraine, Russia, Moldova, Belarus, Uzbekistan, Estonia, and Latvia), education, employment, and family status. Most participants had academic or post-secondary education and were employed across a range of occupational sectors, including education, engineering, social services, finance, and public administration. Data for this study were drawn from a broader qualitative project examining experiences and adaptation among immigrants who arrived during adolescence. Participants' sociodemographic characteristics are presented in Table 1.

**Table 1:** Sociodemographic characteristics of study participants (*N* = 21).

Pseudonym	Gender	Marital Status	Country of Origin (FSU)	Year of Birth	Age at Immigration	Education	Employment
Marina	F	Married	Ukraine	1980	12	Academic	Security
Maxim	M	Married	Ukraine	1977	14	Post-secondary (non-academic)	Technology
Mikhail	M	Married	Russia	1984	15	Post-secondary (non-academic)	Education
Vladimir	M	Married	Moldova	1981	17.5	Academic	Public sector
Ludmila	F	Married	Belarus	1985	13	Undergraduate (ongoing)	Retail
Maya	F	Married	Uzbekistan	1978	12	Academic	Finance
Artium	M	Married	Russia	1985	15	High school	Service occupation
Olga	F	Married	Russia	1982	12.5	Academic	Social services
Roman	M	Married	Russia	1988	12.5	Academic	Engineering
Alina	F	Married	Moldova	1983	13	Academic	Education
Veronika	F	Married	Ukraine	1976	16	Academic	Administration
Karina	F	Married	Moldova	1978	13	Academic	Education
Kiril	M	Married	Estonia	1981	13	High school	Industry
Yuri	M	Married	Ukraine	1978	12	Post-secondary (non-academic)	Transportation
Irina	F	Divorced	Russia	1978	14.5	High school	Administration
Larissa	F	Married	Russia	1980	13	Academic	Education
Svetlana	F	Married	Ukraine	1976	16	Academic	Social services
Galina	F	Married	Ukraine	1976	14.5	Post-secondary (non-academic)	Finance
Anton	M	Single	Estonia	1981	16	Post-secondary (non-academic)	Service occupation
Nikita	M	Married	Russia	1977	14	Academic	Engineering
Pavel	M	Married	Latvia	1983	12	Academic	Technology

### Recruitment

Participants were recruited using a combination of convenience and snowball sampling. Initial participants, identified through social media and personal networks of the researchers and research assistants, referred additional individuals who met the inclusion criteria. Recruitment continued until no new themes emerged from the data, indicating data saturation (Padgett, 2017).

### Data collection

In-depth semi-structured interviews were conducted in 2023 by two research assistants, both fluent in Russian. One held an MSW degree, and the other an MA in migration and social integration. Interviews were conducted in Hebrew, according to participants' preference, with occasional use of Russian expressions.

Interviews lasted between 60 and 90 min, were audio-recorded with participants' consent, and transcribed verbatim. The interview protocol included questions about participants' memories of life before, and early experiences following immigration; challenges encountered during adolescence; and factors that facilitated coping and adaptation over time. Particular attention was given to participants' experiences within institutional settings, including schools, community frameworks, and other social environments.

Interviews encouraged retrospection, inviting participants to reflect back on their experiences during adolescence. While interviews addressed a range of immigration-related experiences, the present study focuses specifically on participants' descriptions of their encounters with institutions.

### Data analysis

Data analysis followed an inductive thematic approach (Creswell, 2013; Patton, 2014). The process began with repeated readings of the transcripts to achieve familiarity with the data. Initial coding was conducted manually, identifying segments related to participants' experiences of immigration, institutional encounters, and coping processes.

The research team, comprising three researchers with backgrounds in social work and migration studies, collaboratively coded an initial set of interviews to develop a shared framework. Through iterative discussions, codes were grouped into preliminary sub-themes, which were later developed into broader themes. Attention was given not only to recurring patterns but also to divergent accounts, in order to capture variation in participants' experiences. Following this stage, one researcher continued the analysis independently, applying and further refining the coding scheme across the full dataset.

Themes were developed through a process of constant comparison within and across cases, ensuring both internal coherence and differentiation. The analysis was guided by an interpretive approach, focusing on how participants made sense of their early institutional experiences. The final themes were defined to capture the core patterns in participants' interpretations of these experiences.

### Ethics

The study was approved by the Ethics Committee of the Faculty of Social and Community Sciences at Ruppin Academic Center (Approval No. 110). All participants received written and verbal explanations about the study and provided informed consent. Confidentiality was ensured through the use of pseudonyms and the removal of identifying details. Audio recordings and transcripts were stored securely and were accessible only to the research team.

### Trustworthiness and reflexivity

Several strategies were employed to enhance the study's rigor (Creswell, 2013; Padgett, 2017; Patton, 2014). Data collection followed a systematic protocol, and the analysis involved both collaborative and independent stages to support intersubjectivity. Regular discussions among the research team enabled the consideration of alternative interpretations and refinement of emerging themes.

The findings are presented with supporting interview quotes, allowing readers to evaluate the interpretations. An audit trail documenting analytic decisions was maintained throughout the research process.

The research team's professional and academic experience informed the interpretation of the data. Reflexive awareness of the researchers' positionality was maintained throughout the study.

## Findings

Analysis revealed four interrelated themes describing participants' retrospective accounts of their experiences with public institutions following immigration. The themes address limited institutional guidance, experiences of misfit and misrecognition, the mediating responsibilities assumed by immigrant adolescents within their families, and localized experiences of support and opportunity.

Table 2 summarizes the main themes and sub-themes.

**Table 2:** Thematic summary of participants' institutional experiences.

Theme	Sub-theme	Short explanation
1. “You're on Your Own”: Absence of Institutional Guidance	1.1. Pre-migration institutional expectations	Participants arrived with prior expectations of institutions, combining distrust with expectations of strong state involvement.
	1.2. Lack of accessible information and guidance	Information and services were perceived as formally available but not clearly communicated or proactively provided. Responsibility for locating information and navigating the system was experienced as falling on the individual, generating feelings of isolation and frustration.
	1.3 Delayed awareness of rights	Rights and services were sometimes discovered only years after migration, sometimes too late. This contributed to feelings of missed opportunities and injustice.
2. Institutional Misfit and Misrecognition	2.1. Educational misplacement and tracking	Language difficulties were interpreted as lack of ability, leading to placement in mismatched tracks (e.g., grade repetition or lower-level grouping), undermining self-worth and perceived competence.
	2.2. Non-recognition of parental skills and cultural capital	Their parents' skills were not recognized or translated into employment opportunities, resulting in status loss, underemployment, and a sense of wasted potential, which participants described as affecting the family as a whole.
	2.3. Cultural insensitivity and stigmatizing attitudes	Institutional representatives were described as displaying dismissiveness, cultural erasure, or stigmatizing attitudes, deepening feelings of alienation and mistrust.
3. Becoming the Family's Institutional Mediators	3.1. Language and bureaucratic mediation	Adolescents acted as translators and technical mediators between their parents and institutions, managing basic interactions with the system.
	3.2. Navigating and managing institutional processes	Adolescents assumed broader responsibilities, including decision-making, understanding rights, and managing financial and health-related matters, compensating for the absence of institutional guidance.
4. Localized Experiences of Support and Opportunity	4.1. Interpersonal recognition and support	Positive experiences were associated with institutional actors (e.g., teachers, commanders, officials) who went beyond formal roles and provided personal, supportive engagement.
	4.2. Institutional opportunities for advancement	Frameworks such as higher-education programs and targeted support initiatives were described as enabling social and economic mobility and creating opportunities for advancement.

Participants recalled early institutional encounters marked by confusion, limited guidance, and misrecognition. Their accounts also described the responsibilities assumed by adolescents in helping their families navigate unfamiliar systems and the specific individuals and institutional programs that provided meaningful support. The following sections present these four themes.

### 1. “You're on Your Own”: Absence of Institutional Guidance

Participants' accounts indicated a perceived lack of institutional guidance in the initial stages following immigration. They described receiving little accessible information, clear orientation, or proactive support from the institutions they encountered. Participants reported having to make sense of and navigate unfamiliar institutional systems independently, often without the knowledge or direction they felt they needed. Across their accounts, the absence of guidance emerged as a central feature of how they remembered and evaluated their early institutional encounters.

#### 1.1. Pre-immigration institutional expectations

Participants often began their accounts by describing the institutional assumptions they brought with them from the Soviet Union. These assumptions combined distrust of institutional actors with expectations regarding the state's responsibility for organizing key aspects of individuals' lives. Participants described these prior institutional orientations as shaping the way they initially approached public institutions in Israel.

Participants described institutional representatives in the Soviet context as extensions of the state rather than supportive figures. Maxim (immigrated at age 14 from Ukraine; all names are pseudonyms used to protect participants' anonymity) explained how this perception shaped his distrust of teachers:

I never trusted teachers, probably already from school in Ukraine. A teacher was a representative of the regime. People in the Soviet Union generally didn't like turning to the authorities, and it was the same with teachers. I never felt this was someone who would help me. On the contrary, it felt like someone who was against me.

He noted that this perception persisted after immigration: “I think it just stayed with me … I never felt like I wanted to approach a teacher with personal problems.”

Similarly, Yuri (immigrated at age 12 from Ukraine) described experiences of humiliation and arbitrary punishment by a teacher in Ukraine, reinforcing his view of institutional figures as hostile and untrustworthy. Upon reflection, he believed that this background had led him to adopt a predominantly cautious and suspicious stance toward authority figures.

At the same time, participants described growing up in a system in which the state played an active role in organizing important aspects of everyday life, particularly education and employment. Against this background, several participants recalled expecting clearer institutional direction following immigration to Israel.

Kiril (immigrated at age 13 from Estonia) expressed disappointment at the absence of such direction: “There was no one pushing me. If I had someone during those years … it would have helped me a lot.” Irina (immigrated at age 14.5 from Russia) similarly described arrival in Israel as a transition into an unstructured environment: “It felt like they put me in a hole and just left me there.”

#### 1.2. Lack of accessible information and guidance

Participants described a lack of accessible and structured information, emphasizing that knowledge regarding existing rights, services, and institutional procedures was not clearly communicated or proactively provided. They often recalled being unsure where to find relevant information, how to interpret it, or how to use it during the initial period following immigration.

Maya (immigrated at age 12 from Uzbekistan) described the lack of guidance within the education system and its broader implications: “They didn't explain what the different types of schools meant… There was nothing accessible … no help at all giving our parents some kind of guide to help them understand which school to choose.” She noted that this lack of guidance extended to everyday aspects of adjustment: “Even basic things: where to shop, what clothes to buy… there should have been something to guide them, instead of setting them up to fail.”

Similarly, Nikita (immigrated at age 14 from Russia) described a context in which information was not shared in an accessible way: “In Israel, somehow it's not customary to share information. The information exists, but it's your job to go and find it.” He described a school placement process that required independent navigation: “My mother had to go on her own to the Ministry of Education… Then we had to go to each [school] ourselves and try to figure out what the options were.”

Participants also pointed to the absence of proactive support, even in situations of significant need. Marina (immigrated at age 12 from Ukraine) described a lack of awareness regarding available support services during a period of family crisis: “There was absolutely no awareness that there were professionals who could support a family, like social workers, or school support teams. No one told us, no one talked to us.”

Kiril (immigrated at age 13 from Estonia) situated these experiences within the limited informational environment participants encountered during the 1990s: “Back then, we had nothing. There was no one to ask, we knew nothing, so it was not an easy period. Many people lost their way.”

Across these accounts, the central difficulty was the gap between available information and participants' ability to locate and use it when needed. This placed the burden of institutional navigation on individuals and their families.

#### 1.3. Delayed awareness of rights

Participants reported that awareness of rights and existing services often emerged only years after immigration. As adults looking back, they described realizing that support had been available but had not been brought to their attention when it was needed. This delayed awareness contributed to a strong sense of missed opportunity and injustice.

Galina (immigrated at age 14.5 from Ukraine) described how her family coped on their own during a profound family crisis following her father's suicide. Only many years later did they learn that support had been available: “Only after more than 20 years, someone contacted us … they told us we had been entitled to psychological support. No one explained anything. Only after all those years did anyone reach out.” Looking back, Galina understood her family's experience differently. What had once been experienced as coping alone was now understood as a situation in which support had existed but had never reached them.

A similar perspective emerges in Alina's account (immigrated at age 13 from Moldova), who now works in education. She reflected on her own experience:

I really try to make things accessible today… to explain, to mediate, to help. … I wish all the support that exists today had existed back then. If I had received that kind of support, I would have reached a different and better place.

Her experience continued to shape the way she approached her professional role, motivating her to provide the guidance and mediation she felt had been missing during her own immigration.

In these accounts, later awareness of support that had not been visible or accessible at the time intensified participants' sense of missed opportunity and loss.

### 2. Institutional Misfit and Misrecognition

Participants reported experiences in which institutional decisions and practices were experienced as poorly aligned with prior knowledge, personal context, and individual potential, giving rise to feelings of injustice, frustration, and diminished self-worth. Three interrelated patterns emerged: educational misplacement and tracking; non-recognition of parental skills and cultural capital; and cultural insensitivity and stigmatizing attitudes by institutional actors.

#### 2.1. Educational misplacement and tracking

Participants reported that educational placement was often determined by language proficiency rather than knowledge or ability. School practices were experienced as arbitrary and, in many cases, deeply unjust, particularly when they resulted in grade retention or placement in lower academic tracks.

Larissa (immigrated at age 13 from Russia) described an automatic policy of grade retention:

There was this completely absurd trend of automatically placing immigrant children a year behind… they put me in sixth grade instead of seventh, just so it would supposedly be easier for me. That was a terrible idea, because a 13-year-old and 12-year-olds are in completely different places mentally.

Similarly, Olga (immigrated at age 12.5 from Russia) described being placed below her age group due to language limitations: “I didn't like that they decided that because I didn't know the language, I had to go a year and a half back… to start in fifth grade instead of seventh. What is that nonsense? Who even asked me?” She noted that this decision was made without her input, delayed her graduation, and caused her to feel out of sync with her peers. Despite differences in the placement decisions they described, both participants recalled them as overlooking the social and developmental implications of separating them from their age peers.

Participants also described being tracked into lower academic levels despite strong abilities. Alina (immigrated at age 13 from Moldova) recounted how language barriers were interpreted as incompetence:

They placed me in lower-level groups in math and English, even though I didn't have a problem with the subjects themselves. The issue was that I didn't understand Hebrew. Instead of helping me, they just lowered my level… from being an excellent student, I suddenly became a failing one.

In other cases, misalignment took the form of accelerated placement without adequate support. Based on assessments indicating advanced knowledge, Artium (immigrated at age 15 from Russia) was placed ahead of his grade level: “They put me straight into tenth grade… They basically said, ‘manage on your own.' I didn't understand anything in subjects like history or Bible. At some point, I left.”

Whether participants were placed below or above their academic level, they described placement decisions as relying primarily on language proficiency while giving limited consideration to their broader educational needs or the support required to succeed.

#### 2.2. Non-recognition of parental skills and cultural capital

Participants reported a mismatch between their parents' qualifications and post-immigration employment opportunities. They described pre-immigration education, skills, and occupational experience as receiving little recognition or not being translated into meaningful employment. Parents' experience of economic marginalization and disruption of professional identity and status affected the entire family. Participants reflected on the emotional impact of witnessing their parents' downward occupational mobility and the practical responsibilities that often accompanied it.

Irina (immigrated at age 14.5 from Russia) described the sharp decline in her parents' professional status after their arrival in Israel: “My father had a master's degree in engineering, my mother was an accountant… But because of the language… it was simply lost. They started with cleaning jobs… He felt lost and devalued.”

Similar experiences were described by other participants, who viewed them as reflecting a broader failure to recognize and utilize their parents' skills and professional experience. Artium (immigrated at age 15 from Russia) linked this to institutional shortcomings: “There were doctors… but because of the language they couldn't validate their credentials… they ended up in cleaning jobs. I think it's simply unacceptable to treat people like that… No one guided them or helped them move forward.”

Participants described their parents' occupational decline as extending beyond employment itself. The loss of professional status shaped family life and reinforced a lasting sense of unrealized potential.

#### 2.3. Cultural insensitivity and stigmatizing attitudes

Participants described encounters with institutional actors marked by a lack of cultural sensitivity and dismissive or degrading attitudes. These interactions, often in vulnerable settings such as schools or health facilities, were experienced as moments in which identity and dignity were undermined, and personal circumstances overlooked.

Olga (immigrated at age 12.5 from Russia) described how her given name was disparaged at school and replaced without consultation: “The teachers said that ‘Olga' is not an appropriate name for Israel, and that it should be changed… they didn't ask my mother, they simply decided.”

Larissa (immigrated at age 13 from Russia) described a different form of cultural insensitivity at school: “There were some books in Russian in the library, but they wouldn't let me take them. To force me to learn Hebrew.” She found this practice harsh, coercive, and dismissive of her cultural background. Although these accounts describe different situations, both participants recalled school practices that they experienced as disregarding important aspects of their linguistic and cultural identities.

In more acute contexts, participants described encounters within the healthcare system as lacking empathy and sensitivity. Marina (immigrated at age 12 from Ukraine) reflected on a painful interaction involving her family during her mother's serious illness:

My grandfather begged the doctor to help save her… he even offered him money, not a lot, but it was all that we had… and the doctor laughed at him. It felt like no one really cared what was happening to us.

Marina explained that, in line with practices familiar to her family before immigration, her grandfather offered the doctor money in a desperate effort to secure further help for her mother. Looking back, she understood that this gesture conflicted with accepted norms in the Israeli healthcare system, yet the doctor's reaction remained a painful memory of humiliation and disregard.

Participants remembered these encounters as experiences in which they felt their identities, cultural backgrounds, or family circumstances were insufficiently understood or respected. These experiences contributed to enduring feelings of alienation and distrust toward institutional actors.

### 3. Becoming the Family's Institutional Mediators

Participants reported assuming responsibility for mediating between their families and institutional frameworks from an early age. Children and adolescents played a central role in navigating their families' engagement with institutions, extending beyond translation to include explanation, guidance, and interaction with personnel. Two interrelated patterns emerged: language and bureaucratic mediation, and navigating and managing institutional processes.

#### 3.1. Language and bureaucratic mediation

Participants reported taking on a central role in translating and mediating everyday interactions between their parents and institutions, including reading and explaining official documents, making phone calls, and accompanying parents to appointments. These tasks were described as routine and expected, despite their complexity.

Galina (immigrated at age 14.5 from Ukraine) and her sister became the primary communicative channel for their parents. Her statement captured the normalization of this role: “My sister and I learned Hebrew faster, and we became our parents' voice. That's it.”

Olga (immigrated at age 12.5 from Russia) described the ongoing nature of this mediation:

“My parents would receive letters and not understand what was written, and I had to call, translate, schedule appointments, and take care of everything.”

Veronika (immigrated at age 16 from Ukraine) described how this mediating role continued into adulthood when assisting her mother:

In most places where I need to help my mother, they speak only Hebrew, and they say explicitly, ‘A response will be provided only in Hebrew.' If a person does not know Hebrew, they are lost.

Her fluency in reading, writing, and speaking Hebrew enabled her to manage these interactions on her mother's behalf.

Marina (immigrated at age 12 from Ukraine) reflected on the impact on family dynamics:

“In a way, it blurs the fact that the parent is supposed to be the dominant figure.”

Language mediation extended well beyond translation. Participants described becoming the primary link between their families and public institutions, while also recognizing how this responsibility altered established family roles.

#### 3.2. Navigating and managing institutional processes

Participants reported deeper involvement in institutional mediation, extending to decision-making, managing procedures, and navigating complex systems on behalf of their families. At a young age, they handled financial, legal, and administrative matters typically associated with the responsibilities of adults.

Alina (immigrated at age 13 from Moldova) described her early assumption of adult responsibilities: “Very quickly, I became the responsible adult for my parents… I was involved in everything: mortgages, foreign currency, rent, reading contracts.”

Maya (immigrated at age 12 from Uzbekistan) described how routine family management became her responsibility:

Basically, all the day-to-day management started falling on me: making appointments at the local health clinic, running around, checking things… I knew how things worked… my mother didn't know many things that I did.

These accounts revealed role reversals, as children became more institutionally conversant than their parents. Irina (immigrated at age 14.5 from Russia) reflected on her central role within the family: “I was the support… I was the one making decisions.”

Participants described becoming the family members most familiar with institutional procedures. This knowledge positioned them as the primary navigators of interactions with public institutions, despite their young age.

### 4. Localized Experiences of Support and Opportunity

Alongside accounts of institutional barriers, participants also described experiences in which they encountered meaningful support. These experiences were typically associated with specific people, programs, or institutional frameworks that provided recognition, guidance, or opportunities for advancement. These encounters were described as exceptions within participants' broader accounts of institutional barriers. Two interrelated patterns emerged: interpersonal recognition and support, and institutional opportunities for advancement.

#### 4.1. Interpersonal recognition and support

Participants recalled specific individuals within institutional settings who responded with flexibility, recognition, and personal engagement. These encounters were experienced positively because participants felt understood, supported, and personally acknowledged.

Ludmila (immigrated at age 13 from Belarus) recalled a teacher who provided ongoing emotional and social support: “He explained things … was interested in our lives … and didn't let go.” She described a sustained engagement beyond his professional responsibilities.

Olga (immigrated at age 12.5 from Russia) described achieving recognition during her national service, in contrast to her earlier school experience: “My manager told me, ‘Olga, stop erasing yourself and use your real name. You deserve to live with your name.'… that's where I understood that I want to become a social worker, like her.” The recognition she received extended beyond that specific encounter, influencing both her sense of identity and her future professional aspirations.

A rare example of bureaucratic responsiveness was described by Vladimir (immigrated at age 17.5 from Moldova), who was conversant in Romanian:

I went to the Ministry of Absorption… I found someone who spoke Romanian. She told me, ‘Write a letter, we'll try to help…Write in Romanian… write what you feel.'… after about a week, I received financial support.

Being able to communicate transformed the encounter from a bureaucratic interaction into one in which he felt understood and supported.

Anton (immigrated at age 16 from Estonia) described the role of his military commander and welfare officer, expressing how actions carried out by committed persons provided both material support and emotional solace: “My commander and the welfare officer really helped me… they made sure I had what I needed, even basic things for my family. I felt that someone actually saw me.”

These accounts suggest that positive institutional experiences were often mediated through individuals rather than systems, emerging through personal engagement, attention, and recognition.

#### 4.2. Institutional opportunities for advancement

Participants identified specific institutional frameworks as providing opportunities for educational and social advancement. Despite early encounters characterized by barriers and misalignment, these programs provided access to resources, recognition, and support.

Maxim (immigrated at age 14 from Ukraine) described how a local absorption coordinator directed him toward a boarding school: “There was an absorption coordinator … he recommended that we apply … he even took us there himself by car. In retrospect, I'm grateful to him, he influenced many families, he really tried to help.” Maxim pointed out how the boarding school facilitated his social inclusion: “The boarding school really helped me… it took me out of what you could call the Russian ‘ghetto' … it gave me a strong step toward integration.”

Svetlana (immigrated at age 16 from Ukraine) described her experience in a university preparatory program as a particularly positive period: “I went to a preparatory program for new immigrants … that was a really nice and enjoyable period… I remember the social life more than the studies.”

Similarly, Veronika (immigrated at age 16 from Ukraine) described how institutional material support provided her a pathway to higher education and advancement opportunities: “There was funding from the absorption center… some kind of financial support they transferred to the university so I could study… I didn't have to pay for the dorms… that's where you felt actual support.”

Unlike support that depended on individual discretion, these structured frameworks created clearer pathways to educational advancement and social integration.

## Discussion

The aim of this study was to examine how individuals who immigrated to Israel during adolescence, often referred to as the “1.5 generation”, retrospectively describe and interpret their early encounters with public institutions. The findings point to a complex and uneven set of institutional experiences. Participants described early institutional encounters as characterized by a lack of accessible information, guidance, and mediation, alongside experiences of institutional misfit and misrecognition. At the same time, they identified more positive experiences in specific institutional contexts, often linked to particular individuals or structured frameworks that provided support and opportunities. Their encounters were also shaped by the institutional orientations they brought with them and by the mediating roles they assumed between their families and formal systems.

A central finding concerns the gap between the formal availability of institutional support and participants' ability to access and use it in practice. Participants' accounts did not point primarily to the absence of services, but to difficulties in accessing and using them, particularly due to limited mediation and guidance. Information regarding rights and available services was not communicated in ways that enabled its use at critical moments, and was sometimes discovered only years later. These experiences were associated with practical disadvantage and a sense of missed opportunity.

The findings therefore draw attention to how institutional support becomes usable in practice through timely information, mediation, and clear routes to services. This insight aligns with literature emphasizing the role of responsiveness in shaping institutional experiences and trust (Kogan and Shen, 2019; Röder and Mühlau, 2012), and indicates that formal provision alone may be insufficient when people do not know how to reach the support available to them.

Participants' evaluations of institutional encounters were shaped by institutional practices as well as by the expectations and prior beliefs they brought with them. In the Israeli case, the role of pre-existing institutional orientations is particularly relevant. This perspective can be understood in light of Van de Walle and Bouckaert's (2003) argument that the relationship between public service performance and trust in government is contingent and mediated by citizens' prior attitudes toward institutions. From this perspective, evaluations of institutional performance are shaped by service quality, expectations, prior beliefs, and broader images of government.

Extending this insight to the context of immigration, the findings suggest that immigrants approach host society institutions with interpretive frameworks formed in their countries of origin. A growing body of literature indicates that immigrants rely on a “dual frame of reference,” comparing institutional experiences in the host country with those in their country of origin (Röder and Mühlau, 2012), and recalibrating their expectations through exposure to different governance systems (Erden and Özçürümez, 2025).

Participants' accounts show how these prior orientations shaped the meaning attributed to institutional encounters in Israel. Participants described a combination of distrust of institutional actors and expectations of strong state involvement in structuring education, employment, and other areas of everyday life (Kagan, 2021; Remennick, 2007). These orientations shaped how the absence of guidance and proactive support was experienced in their institutional encounters.

When institutional guidance was limited, responsibility for navigating unfamiliar systems was often shifted to immigrant families and, in many cases, to the adolescents themselves. Their role extended beyond translation to explaining information, managing bureaucratic procedures, arranging services, and participating in decisions on behalf of their families. They therefore became central intermediaries through whom their families accessed and engaged with public institutions, despite still learning the language and becoming familiar with the institutional system themselves.

These findings resonate with existing research on immigrant youth as cultural and linguistic brokers (Katsiaficas, 2017; Suárez-Orozco et al., 2009). Such roles, often associated with the “1.5 generation,” may involve broad and significant responsibilities in interactions with institutions. This form of agency was closely linked to participants' age at immigration and their relatively rapid acquisition of language and cultural competencies.

Beyond issues of access and functionality, the findings highlight the symbolic dimension of institutional encounters. Educational placement decisions were one expression of this dimension. Participants described being placed above or below their expected grade or academic level on the basis of partial assessments that gave insufficient consideration to their linguistic, social, and educational needs. Participants described instances of educational misplacement, non-recognition of parental skills, and culturally insensitive interactions as experiences in which their abilities, identities, and family circumstances were insufficiently recognized. Such encounters were remembered as consequential for participants' sense of dignity, belonging, and inclusion. Consistent with prior research (Belabas and Migchelbrink, 2025; Tyrberg, 2024), perceptions of recognition and fair treatment play a central role in shaping satisfaction. Participants also recalled encounters in which recognition and support were provided, indicating that institutional experiences were not uniformly negative.

Positive institutional experiences were frequently linked to specific individuals (teachers, military officers, or administrators) who provided recognition, flexibility, and support, highlighting the significance of concrete interpersonal encounters. In line with Giddens' (1991) concept of “access points,” these interactions constituted important points through which participants experienced and evaluated institutions. These experiences stood out in their accounts because they did not characterize most of the institutional encounters they described.

Alongside these interpersonal encounters, participants identified structured institutional frameworks, such as educational programs and targeted support initiatives, as important sources of support. These experiences can be understood as “pockets of support” within a broader range of institutional encounters. Such frameworks provided access to resources, recognition, and opportunities for advancement.

The four themes show that participants' evaluations of formally available institutional resources were shaped by how accessible and usable those resources were perceived to be. Timely guidance, mediation, recognition, and support were central to these evaluations. When these were perceived as absent, inadequate, or poorly aligned with participants' needs and circumstances, families were required to bridge the gap between formal provision and practical accessibility, often through the adolescents themselves. Participants' prior institutional orientations shaped how this gap was experienced. Used as an analytical lens, the retrospective perspective made it possible to examine how participants understood their early institutional encounters and their significance from the broader vantage point of adulthood.

### Practical implications

The findings suggest several implications for policy and practice. First, the perceived lack of accessible information and guidance underscores the importance of proactive institutional communication, particularly during the early stages of immigration. Enhancing visibility and usability of services at the relevant time may reduce reliance on informal mediation and prevent missed opportunities. Second, the findings highlight the importance of recognition and cultural sensitivity in institutional encounters. Training frontline professionals may improve their ability to engage with diverse populations in ways that promote both accessibility and dignity. Third, strengthening systems that provide structured guidance and help, particularly in educational and transitional frameworks, may contribute to social integration and perceptions of institutional support. This is especially the case when such settings combine access to resources with experiences of recognition and inclusion. Fourth, there is a need to recognize that for immigrants arriving during adolescence, institutional settings such as schools are crucial for providing opportunities and shaping how support is accessed and understood. The findings further suggest the importance of reducing reliance on adolescents as intermediaries by offering their families explicit guidance on navigating systems.

### Limitations and future research

This study draws on retrospective accounts, reflecting how participants currently interpret past experiences. While this perspective provides valuable insight into processes of meaning-making, it may be shaped by subsequent experiences and changing positions. Future research could combine retrospective and longitudinal approaches to examine institutional experiences and evaluations at different stages following immigration. Participants in this study were predominantly educated, employed, and socially stable at the time of the interviews. Their current positions may have shaped how they interpreted their early institutional encounters, and the accounts of immigrants with less stable educational, occupational, or social circumstances may differ. Participants originated from different former Soviet republics, and the study was not designed to examine systematically how country-specific institutional, linguistic, or cultural contexts shaped their experiences. In addition, the findings are shaped by the specific Israeli context and may not fully generalize to other settings. Finally, in light of their growing availability, future research could examine how digital services for immigrants shape their access to and engagement with institutions (Bielewska et al., 2025).

## Data Availability

The datasets presented in this article are not publicly available due to ethical and confidentiality considerations related to qualitative interview data. Participants were assured that their data would remain confidential, and sharing the data could compromise their anonymity. The data are not available upon request.
